# The Materials Science beamline upgrade at the Swiss Light Source

**DOI:** 10.1107/S0909049513018475

**Published:** 2013-07-16

**Authors:** P. R. Willmott, D. Meister, S. J. Leake, M. Lange, A. Bergamaschi, M. Böge, M. Calvi, C. Cancellieri, N. Casati, A. Cervellino, Q. Chen, C. David, U. Flechsig, F. Gozzo, B. Henrich, S. Jäggi-Spielmann, B. Jakob, I. Kalichava, P. Karvinen, J. Krempasky, A. Lüdeke, R. Lüscher, S. Maag, C. Quitmann, M. L. Reinle-Schmitt, T. Schmidt, B. Schmitt, A. Streun, I. Vartiainen, M. Vitins, X. Wang, R. Wullschleger

**Affiliations:** a Swiss Light Source, Paul Scherrer Institut, CH-5232 Villigen, Switzerland; b Laboratory for Micro- and Nanotechnology, Paul Scherrer Institut, CH-5232 Villigen, Switzerland; c Excelsus Structural Solutions SPRL, B-1150 Brussels, Belgium; d Department of Machine Engineering, Paul Scherrer Institut, CH-5232 Villigen, Switzerland

**Keywords:** beamline upgrade, undulator radiation, X-ray optics, powder diffraction, surface diffraction, coherent X-ray diffraction imaging

## Abstract

The Materials Science beamline at the Swiss Light Source has been operational since 2001. In late 2010, the original wiggler source was replaced with a novel insertion device, which allows unprecedented access to high photon energies from an undulator installed in a medium-energy storage ring. In order to best exploit the increased brilliance of this new source, the entire front-end and optics had to be redesigned. In this work, the upgrade of the beamline is described in detail. The tone is didactic, from which it is hoped the reader can adapt the concepts and ideas to his or her needs.

## Abstract 2

The wiggler X-ray source of the Materials Science beamline at the Swiss Light Source has been replaced with a 14 mm-period cryogenically cooled in-vacuum undulator. In order to best exploit the increased brilliance of this new source, the entire front-end and optics have been redesigned.

## 1. Introduction   

The Materials Science (MS) beamline at the Swiss Light Source (SLS) was originally conceived to provide hard X-rays in the energy range 5–40 keV (Patterson *et al.*, 2005 ▶) and serve experiments in powder diffraction (PD), surface diffraction (SD) and computed tomography. As such, it was the only insertion-device beamline at the SLS to exceed a photon energy of 20 keV.

In 2000, it was only possible to access such high photon energies at the intermediate storage-ring electron energies of 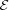 = 2.4 GeV of the SLS by employing a so-called minigap wiggler (called ‘W61’ to indicate the magnets’ period of 60.5 mm), with a critical energy of 7 keV and a deviation parameter *K* = 8.6 at a gap size of 8 mm (Patterson *et al.*, 2005 ▶). The transmitted flux at 12 keV from the wiggler of approximately 10^13^ photons s^−1^ after monochromatization through a Si(111) double-crystal monochromator (DCM) was at best 30% efficient. This was due primarily to the large heat load on the first DCM crystal, which caused a thermal bump and consequent reduction in flux, despite the use of the dynamical correction system ‘TORII’ (Schulte-Schrepping *et al.*, 1995 ▶). Another consequence of the high output power of W61 was the need to use a so-called rotating carbon filter to remove the strongly absorbed soft X-ray part of the wiggler spectrum below approximately 5 keV, amounting to 1.6 kW, a power too high to be able to use a cooled diamond filter. This front-end component, as well as two beryllium windows, increased the effective source size to approximately 450 µm (h) × 250 µm (v).

Since 2000, however, undulator technology has matured, in particular with regards to magnet materials and in-vacuum technology. As described in detail in the next section, high photon energies can begin to be accessed at medium-energy storage rings, once radiation-hard small-period magnets with high maximum magnetic field strength *B*_0_ become available. Such magnets were developed around 2003, and their implementation in undulator technology has allowed a considerable reduction of undulator-magnet period lengths from 19 mm to 14 mm.

With the advent of this technological advance, it was decided in 2007 to replace W61 with an in-vacuum cryogenically cooled permanent-magnet undulator [CPMU (Hara *et al.*, 2004 ▶)], having a magnet period λ_u_ = 14 mm, resulting in the CPMU being called ‘U14’.

Because the beam divergence of U14, and hence the beam cross section at the optics hutch, is so much smaller than that of W61, new optics had to be designed (see §6). At the same time, the new design also took into account the possibility of introducing new techniques at the beamline, such as coherent X-ray diffraction imaging and micro Laue diffraction.

The paper is organized as follows: after a description of the specifications of the SLS storage ring, the U14 undulator and Materials Science beamline given in §2 and §3, details of the beamline layout, the front-end components and optics design are described in §4, §5 and §6, respectively. The performance of the beamline after the upgrade is covered in §7, and the impact of this on the two endstations, plus first results, are described in §8 and §9.

## 2. Specifications for the SLS and the Materials Science beamline   

Since the last publication describing the MS beamline (Patterson *et al.*, 2005 ▶), the storage-ring specifications have improved considerably in terms of vertical emittance and electron-beam size. This is due mostly to an exceedingly low coupling of the horizontal to the vertical emittance of 5 × 10^−4^. Such coupling is caused primarily by slight misalignments of the focusing quadrupole magnets. The most important parameters are listed in Table 1 ▶.

The SLS runs in ‘top-up’ operation, in which the storage current is maintained to within 1% of the nominal value of 400 mA. This feature is important in providing a constant X-ray flux and stable beam position.

The specifications of the MS beamline are summarized in Table 2 ▶. The improved coherent fraction promised by undulator radiation should also be exploited. This feature sets stringent specifications on both the optics and on the vacuum quality; the gradual build-up of carbon deposits on the surface of the first crystal in the DCM, caused by the photo-induced cracking of carbon-containing gaseous species in the residual gas, can rapidly and adversely affect the quality of the wavefront. The residual gas pressure in the DCM was therefore specified as being less than 10^−8^ mbar.

## 3. The U14 undulator   

### 3.1. Theoretical design considerations   

**Figure 1:**
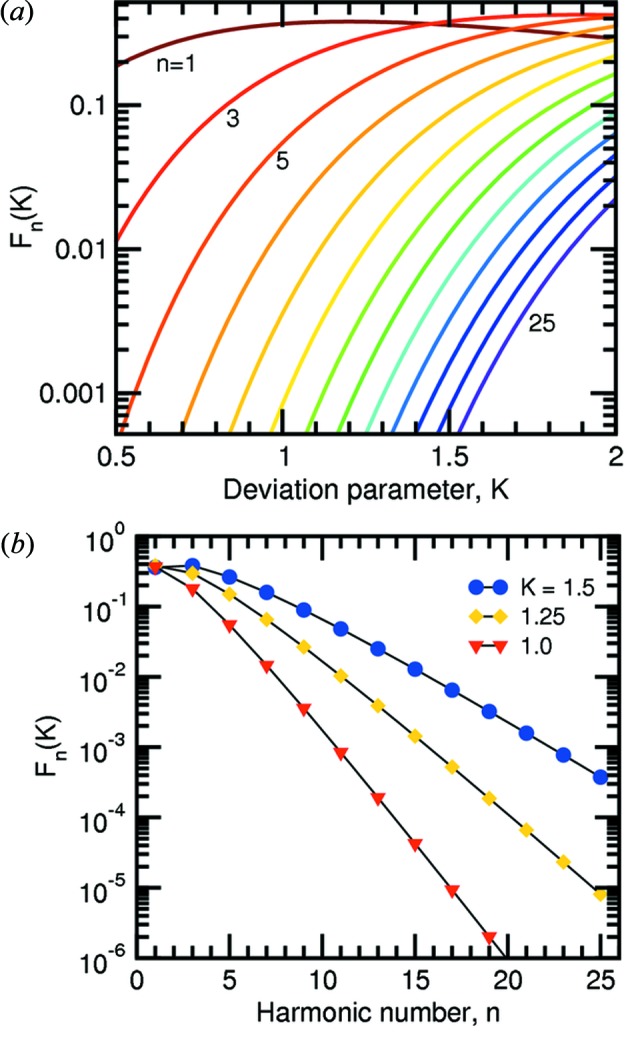
Plot of the tuning function *F*_*n*_(*K*) as a function of (*a*) *K*, for the odd harmonics between *n* = 1 and 25; and (*b*) *n*, for three different values of *K*.

The on-axis peak intensity *I*_*n*_ of the *n*th harmonic of undulator radiation is given by 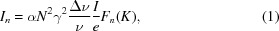
whereby α = 1/137 is the fine-structure constant, γ is the Lorentz factor 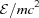, Δν/ν = 1/*nN* is the relative spectral bandwidth of the *n*th harmonic peak, *I* is the current, *e* is the elementary charge, *N* is the number of magnet periods and *F*_*n*_(*K*) is the so-called tuning function, given by 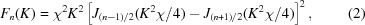
whereby 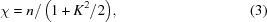
and *J* are Bessel functions of the first kind. *F*_*n*_(*K*) is plotted as a function of *K* in Fig. 1(*a*) ▶. The deviation parameter *K* is defined as 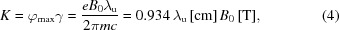
whereby 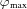 is the maximum angular excursion off-axis of the electrons passing through the undulator magnet array, *m* is the electron rest mass, and *c* is the speed of light in a vacuum (Willmott, 2011 ▶).

One sees immediately from Fig. 1(*a*) ▶ that, even for relatively high values of *K* ≃ 1.5, *F*_*n*_(*K*), and hence also *I*_*n*_, is three orders of magnitude smaller for *n* ≃ 21 than for the first few harmonics. Fig. 1(*b*) ▶ is a plot of *F*_*n*_(*K*) *versus* harmonic number *n* for three values of *K*.

The condition for constructive interference on-axis in an undulator is given by 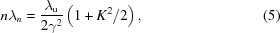 or, in practical units, 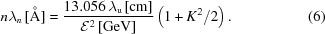
Hence, for a given storage-ring energy 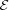, undulator magnet period λ_u_ and deviation parameter *K*, *n*λ_*n*_ is a constant, which, according to Fig. 1(*a*) ▶, we would like to be as small as possible, in order to access those tuning curves with as low as possible value of *n*. On the other hand, we require *K* to be as large as possible (Fig. 1*b* ▶). The only possibility to achieve both these requirements is, therefore, to reduce λ_u_ [equation (6)] and increase *B*_0_ [equation (4)].

The previous shortest-period undulator at the SLS, U19, had λ_u_ = 19 mm. In order for the insertion device to remain an undulator, *K* should not exceed 2.0 or thereabouts. Hence, from equation (4), the maximum magnetic field strength must be equal to or less than approximately 1.1 T. For the maximum *K* of 2.0, *n*λ_*n*_ for U19 was equal to 12.92 Å at the SLS. Accessing photon energies above 30 keV thus required working on the 31st harmonic, for which the intensity is unacceptably low [see Fig. 1(*a*) ▶]. Indeed, U19 at the protein crystallography beamline at the SLS operates only up to 19 keV photon energy.

**Figure 2:**
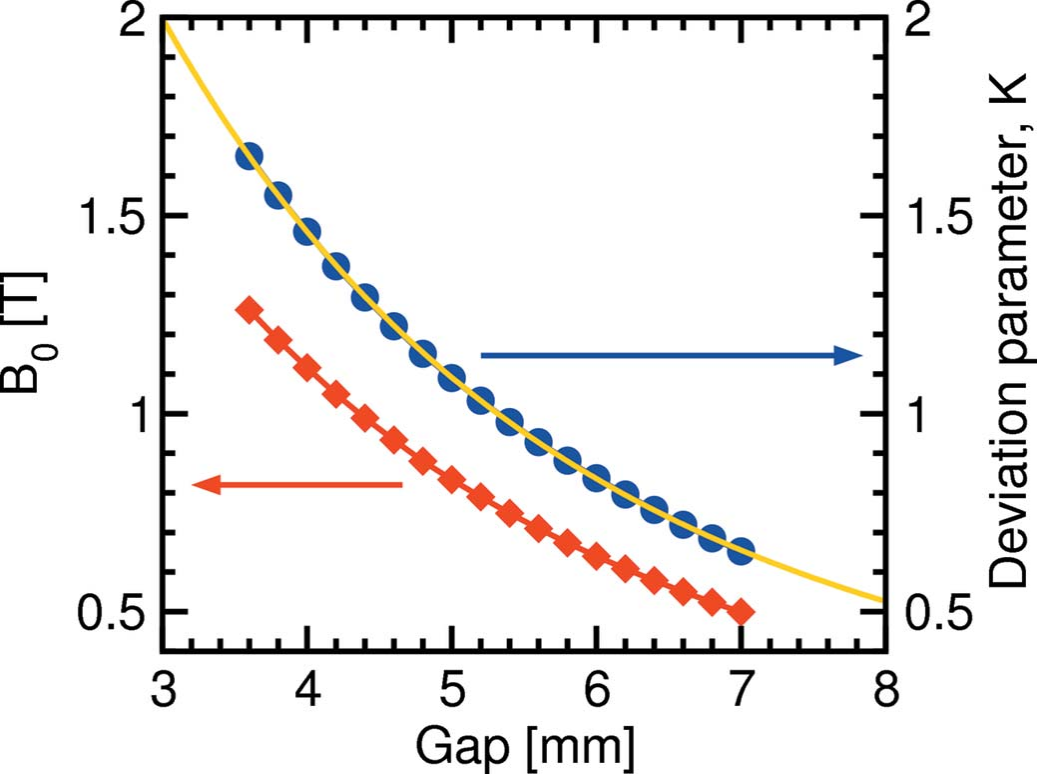
Plot of the maximum on-axis magnetic field *B*_0_ and deviation parameter *K* as a function of the U14 undulator gap size. The yellow solid curve is a best fit to *K* [see equation (7)].

Because the U14 undulator is an in-vacuum insertion device, the gap size can in principle be closed to as little as 3.5 mm and the maximum magnetic field strength *B*_0_ be as large as 1.5 T (Hara *et al.*, 2004 ▶). This was measured as a function of gap size *g* using a Hall probe (see Fig. 2 ▶). The resulting *K* values [equation (4)] were then fit, yielding 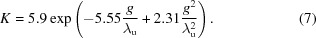
In practice, the minimum gap size has been limited to 3.8 mm.

From equation (4), the maximum value of *K* is approximately 1.65, and *n*λ_*n*_ = 7.49. Accessing 30 keV photons requires one to operate at the 17th harmonic, for which the flux is an order of magnitude higher than that for U19 [see Fig. 1(*a*) ▶].

### 3.2. The U14 CPMU design   

A detailed description of the U14 CPMU design and performance is given elsewhere (Calvi *et al.*, 2013 ▶). Here, the most important features are highlighted.

The undulator design parameters compared with those for the W61 wiggler and U19 undulator are summarized in Table 3 ▶. The magnet material chosen for U14 was Neomax S45SH, which has the alloy composition Nd_2_Fe_14_B. The remanent field of this material maximizes at approximately 140 K (Hara *et al.*, 2004 ▶) and is higher than related commercial magnets. Importantly, demagnetization caused by exposure to high-energy electrons is almost completely suppressed at this temperature. While at room temperature the magnetic field strength drops by almost 30% after exposure to 1.5 × 10^15^ 2 GeV electrons, the same exposure at 140 K produces a drop in field strength of approximately 1%. U14 is hence cryogenically cooled and maintained at 138 ± 0.1 K, by a combination of liquid-N_2_ cooling and heating elements. The temperature is measured at three positions (upstream, centre, downstream) on both the upper and lower undulator support structures. A second important aspect of this material is that, although the magnetic field strength only increases modestly when cooled to 140 K, its coercivity increases by over a factor of two, allowing one to use it at small gap sizes.

The liquid-N_2_ pump system is also used to cool the first crystal of the DCM (see §6.2). This element, including the internal metallic bellows for the nitrogen flow, causes its thermally insulated goniometer stage to cool over approximately 100 h from room temperature to 290 K. This has the sadly unavoidable consequence that experiments at high energy, for which the DCM crystals’ Darwin widths are narrow, are relatively unstable during this cool-down period, as the goniometer slowly drifts thermally.

**Figure 3:**
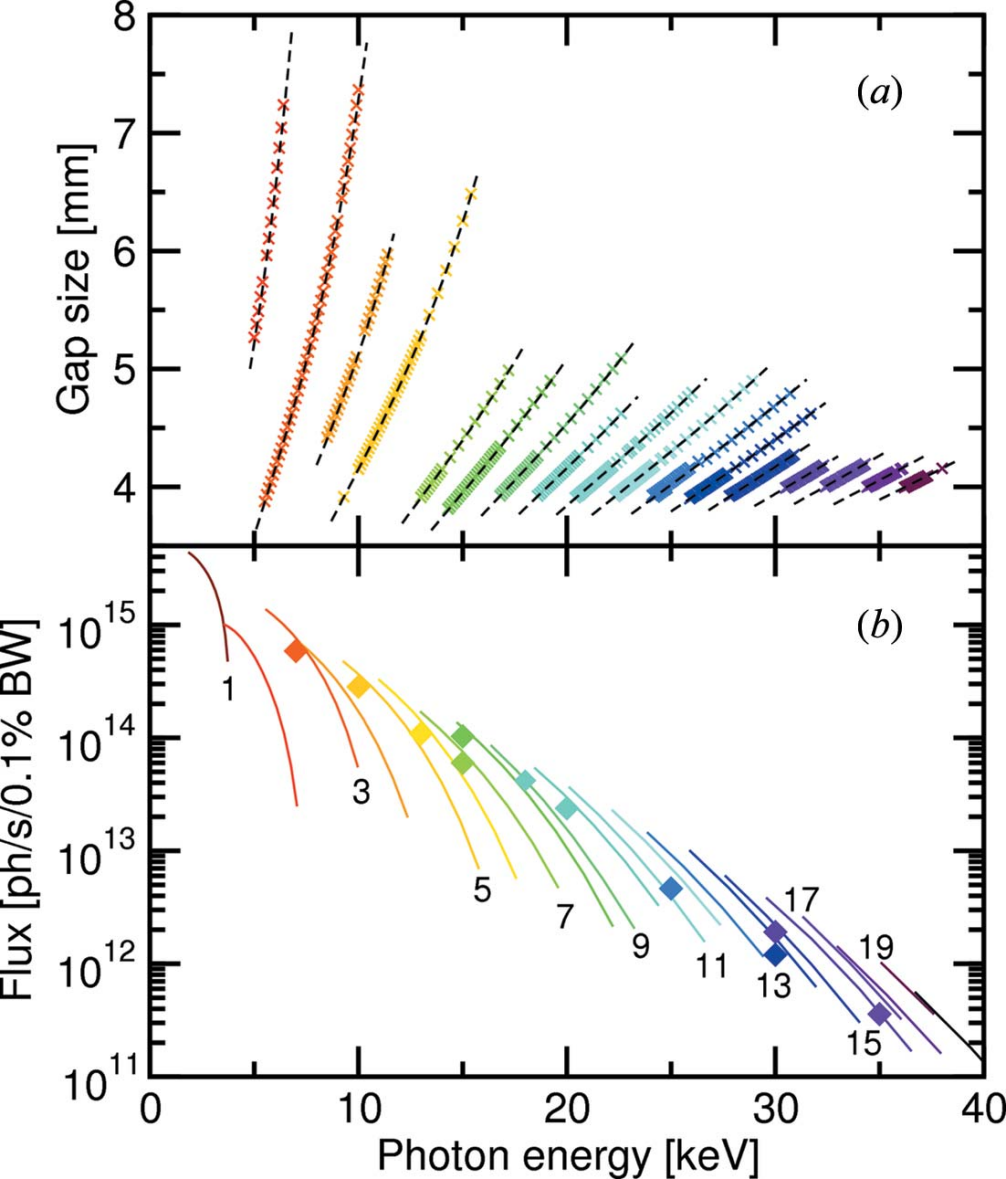
Plots of (*a*) the experimental gap sizes (crosses) as a function of photon energy between the third and 19th harmonics, plus the theoretical curves, as calculated with *SRW* (dashed lines); and (*b*) the theoretical photon flux after monochromatization by the DCM for all harmonics from the first to the 20th, also calculated using *SRW*. The diamond symbols are the experimentally determined fluxes, taking into account all correction factors associated with the beamline components and detector efficiency. Their colours indicate at which harmonic the flux was recorded.

**Figure 4:**
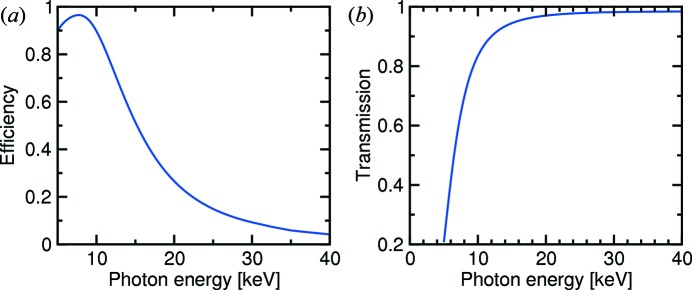
(*a*) Plot of the quantum efficiency of the Pilatus 100k pixel detector used at the SD station, including absorption due to the protective layer of aluminium, mylar and Si_3_N_4_ in front of the detector, over the range 5–40 keV. (*b*) Plot of the transmission spectrum of X-rays through 236 µm of diamond (density = 3.51 g cm^−3^) in the range 5–40 keV.

The short undulator period allows more magnet poles to be installed (*N* = 120, compared with that of U19 at the SLS, for which *N* = 96; see Table 3 ▶). From equation (1), we see that the brilliance of U14 is also enhanced by a factor of approximately 1.5 because of this aspect. Despite the complication of cooling the CPMU, it was possible to maintain a phase error of approximately 2.5° between the useful gap sizes of 3.8–6 mm. Plots of the variation of the gap size with photon energy for the third to 19th harmonics, plus the theoretical flux, taking into account phase errors, are shown in Fig. 3 ▶, calculated using the *SRW* code (Chubar *et al.*, 1998 ▶; see also http://www.esrf.eu/Accelerators/Groups/InsertionDevices/Software/SRW). Also included in the latter are experimentally determined fluxes, recorded at the SD station using the photon-counting Pilatus 100k detector, corrected for the various kapton, diamond, mylar and Si_3_N_4_ components, the detector efficiency [Fig. 4(*a*) ▶], the mirrors’ capture cross-section (a factor which begins to become important above approximately 20 keV) and the bandwidth of the Si(111) crystals in the DCM (see below). At lower energies, the experimentally obtained flux is 70% or better compared with theory, dropping to approximately 50% above 25 keV.

**Figure 5:**
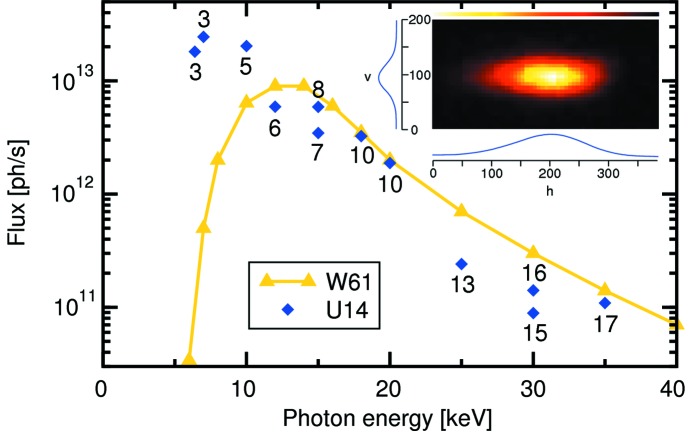
Plot of the experimentally determined flux recorded at the SD station, compared with that provided by the W61 wiggler previously installed at the MS beamline. The data have only been corrected for the Pilatus 100k sensitivity. For each data point the harmonic number at which it was recorded is given. In the inset the beam focus at the SD station at 9 keV is shown. The axes are in micrometres.

Of more direct interest to the user, however, is the actual number of photons per second that he/she can expect after the optics hutch, as a function of energy. This is shown in Fig. 5 ▶. For purposes of comparison, the total flux of the old wiggler insertion device W61 is also plotted. At the lowest photon energies, the flux from U14 is orders of magnitude higher, dropping to about 50–75% of the W61 flux at the highest photon energies. Given that the unfocused beam of U14 is over 200 times smaller than that of W61, the areal flux density of U14 is thus over two orders of magnitude higher.

At the SD station, the best focus is approximately 130 µm (h) × 40 µm (v), or about 20 times smaller than previously obtainable (see the inset of Fig. 5 ▶) recorded at 9 keV.

## 4. Beamline layout   

**Figure 6:**
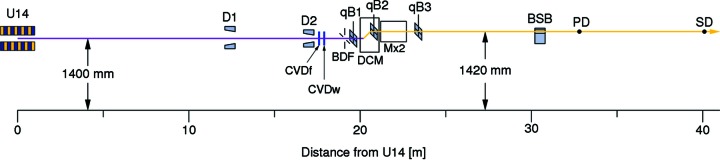
Block diagram of the positions of the most important beamline components. The white/pink beam is shown in magenta, the monochromatic beam in yellow. U14 = U14 undulator; D1 = diaphragm 1; D2 = diaphragm 2; CVDf = CVD filter; CVDw = CVD window; BDF = beam-defining slits; qB1(2,3) = qBPM 1(2,3); DCM = double-crystal monochromator; Mx2 = double mirror chamber; BSB = Bremsstrahlung blocker; PD = centre of powder diffractometer; SD = centre of surface diffractometer.

The positions of the most important beamline components are listed in Table 4 ▶, and shown schematically as a block diagram in Fig. 6 ▶. Most of these, in particular the optics between 20 and 31 m, are described below in detail. It is briefly mentioned here that, because the tomography station was removed from the beamline in 2007, an aspect of the upgrade program was to move the powder diffractometer 3.5 m upstream, while the focusing optics were moved approximately 70 cm downstream, compared with the former configuration. This means that it is now possible to demagnify the photon beam at PD to 0.6, which is beneficial for certain experimental set-ups such as those using diamond-anvil cells, or in micro-Laue experiments.

The surface diffractometer has also been moved 70 cm upstream in the second hutch. Although this has only a marginal impact on the demagnification of the focal spot size, it does allow the possibility of increasing the sample–detector distance to over 2 m. As described in §9.2.1, this becomes important for future possible experiments exploiting coherence, such as coherent X-ray diffraction imaging (CXDI).

## 5. The front-end   

The most important front-end components are listed in Table 4 ▶, from the mask at 8.245 m to the CVD diamond window at 17.851 m. The two-blade X-ray beam-position monitors (XBPMs) allow one to monitor both the height and angle of the white X-ray beam produced by U14. Downstream from these are two water-cooled diaphragms which intercept the soft X-ray outer cone of the synchrotron beam. The transmitted power through the second (narrower) diaphragm is 334 W.

Approximately half of this transmitted power lies in the soft X-ray regime below approximately 5 keV, which interacts very strongly with condensed matter and must therefore be removed to protect the X-ray optical elements. This is achieved with a combination of a 120 µm-thick water-cooled diamond filter, and an 80 µm-thick water-cooled diamond window, after which the transmitted power is 157 W. The transmission spectrum of 236 µm of diamond is shown in Fig. 4(*b*) ▶. The additional 36 µm of diamond is included in order to account for the three quadrant CVD-diamond beam-position monitors (qBPMs) permanently positioned in the beam in the optics hutch (Schulze-Briese *et al.*, 2001 ▶).

## 6. Optics design concept   

### 6.1. Choice of configuration   

**Figure 7:**
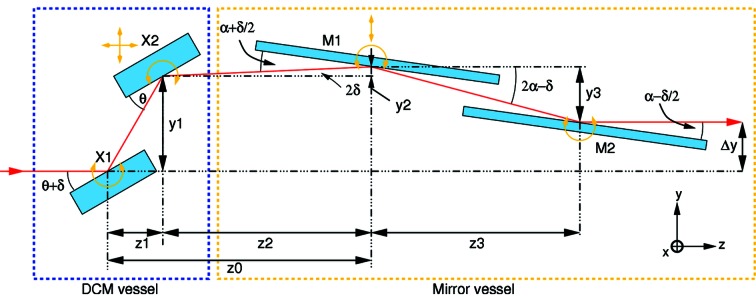
Schematic figure of the optics set-up. Movements required of each of the four components (crystals X1 and X2, and mirrors M1 and M2) are shown by yellow arrows. The relevant dimensions used in calculating the optics elements’ positions are also given. The beam offset after exiting the mirror chamber is Δ*y* = 20 mm.

Two mirrors are necessary to sufficiently suppress harmonic contamination (Patterson *et al.*, 2005 ▶). The previous optics configuration with W61 was collimating-mirror–DCM–focusing-mirror. As such, the first mirror (1 m long) absorbed as much as 1200 W of power from the wiggler source and therefore required water cooling (Patterson *et al.*, 2005 ▶). There are some important advantages in replacing this set-up with one in which the two mirrors are downstream of the DCM (*i.e.* DCM–mirror–mirror, see Fig. 7 ▶). These include not needing to cool the first mirror, plus the option of using only one of the mirrors for experiments which profit from using as few optical elements as possible, such as CXDI. Lastly, in such a configuration, both mirrors can be housed in the same vacuum vessel, making the design more compact and cheaper.

**Figure 8:**
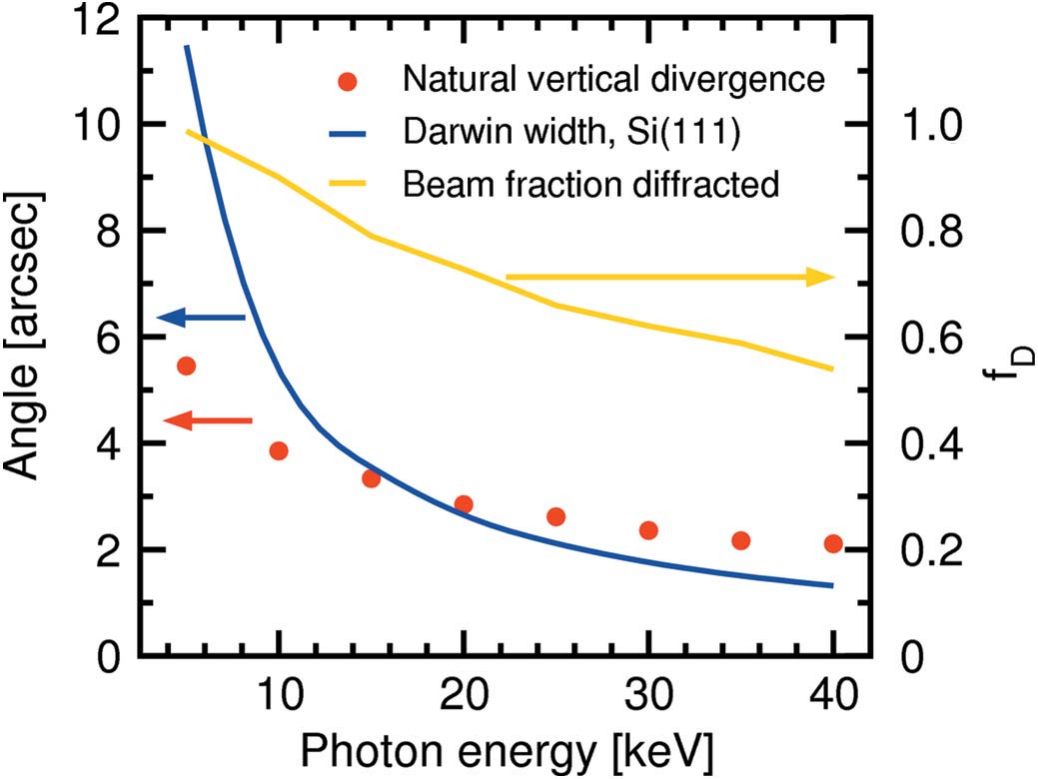
Plot of the theoretical Darwin width and the natural vertical divergence of the monochromatic undulator radiation as a function of photon energy. Also shown is *f*_D_, the beam fraction that lies within the Darwin width and is consequently diffracted.

In reaching a decision as to whether such a change of configuration should be adopted, the relative sizes of the natural vertical divergence (FWHM) of the undulator radiation and the Darwin width of the DCM Si(111) crystals had to be compared. The standard deviation of the undulator radiation perpendicular to the orbital plane is given by 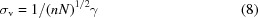
and the FWHM of the beam in this direction is therefore 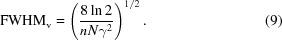
This is plotted in Fig. 8 ▶ along with the theoretical Darwin widths of Si(111) for selected energies. If one makes the simplification that the Darwin curve approximates a top-hat profile of width 2*W*_D_, the fraction *f*_D_ of the beam diffracted by the Si(111) crystal is given by 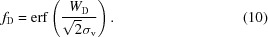
This is also shown in Fig. 8 ▶. Where the two angles overlap (approximately 16 keV), one FWHM of the undulator radiation is intercepted by the Darwin curve, that is, 76% of the flux. At the highest desired energy of 40 keV, almost 50% of the beam intensity is lost. Nonetheless, because the large majority of experiments are conducted at photon energies below 25 keV, the advantages of this new configuration were deemed to outweigh the loss of flux at higher energies.

### 6.2. Detailed geometry   

**Figure 9:**
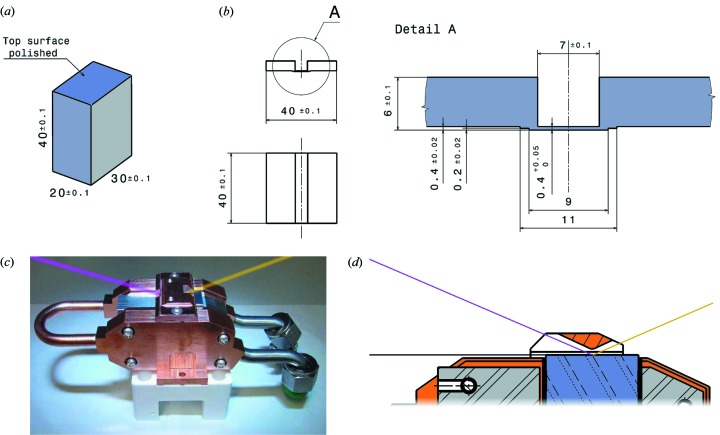
Dimensions of (*a*) crystal 1 (X1), and (*b*) crystal 2 (X2) in the DCM, given in millimetres. (*c*) X1 mounted in the liquid-N_2_-cooling housing. The ‘white beam’ impinges from the left, while the monochromatic beam (here, yellow) emerges on the right. (*d*) The crystal surface itself is obscured by a copper ‘cap’ used to protect it from long-term deposits originating from residual-gas carbon-containing species, by limiting the solid angle to which the surface is exposed. Materials: orange = Cu; grey = Al; blue = Si.

The fixed-exit DCM design is similar to those already used at the protein crystallography beamline PX1 (Schulze *et al.*, 1998 ▶) and the computed tomography beamline TOMCAT (Stampanoni *et al.*, 2007 ▶), both at the SLS. The DCM was designed and built by CINEL s.r.l., Padova, Italy (http://www.cinel.com). Both Si(111) crystals are mounted on their own goniometer. The second crystal can be bent sagittally to provide horizontal focusing (Schulze *et al.*, 1998 ▶) (Fig. 9 ▶). A crucial difference, however, between the new optics design at MS and those at PX1 and TOMCAT is the beam offset (*i.e.* the difference in height between the incoming white beam from the undulator and the outgoing monochromatic beam). At PX1, this is +50 mm. Because at the MS beamline, we wish to access photon energies as high as 40 keV; having a fixed-exit beam with this offset would require the second crystal (X2) to move by approximately 600 mm in the beam-propagation direction (*z*), setting unreasonable specifications on the linear-motion stage for X2 with regards to parasitic motions, and also increase the volume of the monochromator vacuum chamber by a significant fraction. Radiation dosage studies of the Bremsstrahlung radiation at different offsets were performed, from which an offset of +20 mm was deemed to be well within the safety limits and meant that the maximum *z*-translation of X2 is reduced to approximately 200 mm.

**Figure 10:**
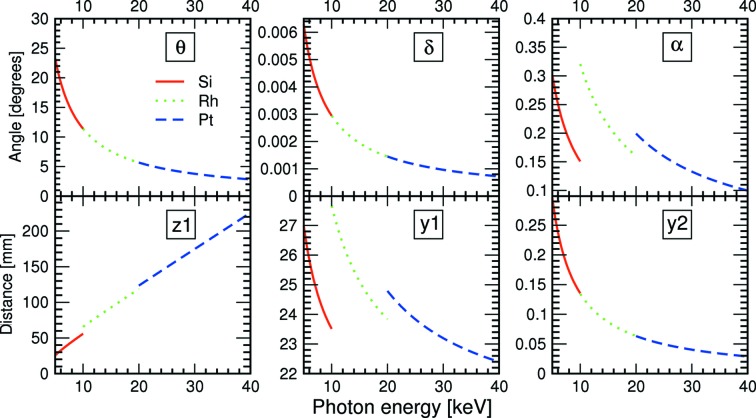
Positions and angles of the optics components as a function of photon energy for the three different mirror-stripe settings.

This smaller offset compared with that of the PX1 beamline meant, however, that we could not take a carbon copy of the two crystal designs used in the PX1 monochromator (Schulze *et al.*, 1998 ▶). The reason for this is that at low photon energies the crystals and their holders would overlap vertically, both occluding the beam and increasing the danger of crashing the crystals into each other when changing the photon energy. As calculated in this section, the vertical separation of X1 and X2 at 5 keV is only approximately 25 mm (see Fig. 10 below), too small to be able to use the same model of the PX1 beamline. The design concept, however, could be preserved, requiring only that the dimensions of X1 and X2 be scaled down. The most important design parameter was that the ratio of the length to width of the thinned bendable section of X2 (0.4 mm thick, used for sagittal focusing) be approximately 6 or greater to avoid anticlastic distortions [see Fig. 9(*b*) ▶].

Another novel feature is shown in Figs. 9(*c*) and 9(*d*) ▶. It is well known that the coherent wavefront of quasimonochromatic synchrotron radiation can degrade over time because of the build-up of carbonaceous deposits on X-ray optics surfaces (especially on those exposed to high-intensity polychromatic beams), due to the cracking of carbon-containing species in the residual gas within the vacuum chamber (Chauvet *et al.*, 2011 ▶). Considerable effort was invested to optimize the DCM vacuum without incurring unacceptable costs associated with ultrahigh-vacuum (UHV) motors and motion stages. An ultimate pressure better than 10^−8^ mbar was specified. This was met by CINEL.

However, the effective incident flux of residual-gas species can be further reduced by an order of magnitude by limiting the solid angle ‘seen’ by the optics. This was achieved on X1 [on which the high-power white beam is incident, Fig. 9(*c*) ▶] by installing a copper ‘cap’ which only opens up to an angle of approximately 25° perpendicular to the crystal surface (Fig. 9*d* ▶). The opening solid angle of the cap is approximately 1 sr, or 16% of an unprotected surface. The first Si(111) crystal X1 and thus also the cap are maintained at liquid-nitrogen temperatures, and the latter therefore acts as a sacrificial cryopump, protecting X1. After more than one year of operation, no sign of a burn spot could be seen.

A schematic diagram of the DCM and mirrors’ configuration is shown in Fig. 7 ▶. The second Si crystal X2 is at room temperature. Because of the temperature difference of the two crystals (Δ*T* ≃ 200 K), they also have marginally different Bragg angles, due to the thermal contraction of silicon in X1.

In order to maintain a constant height for the exit beam after M2 (+20 mm relative to the incoming beam) for all photon energies, the second crystal must be translated both vertically (*y*) and horizontally (*z*), while the first mirror M1 must be translated vertically. In addition, the mirror angles are adjusted with photon energy, in order to minimize harmonic content. Both mirrors have three regions: bare silicon, Rh-coating and Pt-coating. The first mirror is kept flat, while the second mirror M2 can be bent to provide vertical (meridional) focusing.

The thermal expansion coefficient of silicon changes almost linearly between 77 and 300 K (Slack & Bartram, 1975 ▶) such that 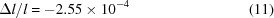
as one cools from 300 to 77 K.

We let the Bragg angle at 300 K be θ for the room-temperature crystal X2, and that for the same photon energy of X1 be θ + δ. Starting from Bragg’s law, it is trivial to demonstrate that 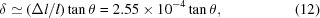
where δ 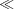 1 is given in radians.

The consequence of this is that the beam is tilted upwards by an angle 2δ after X2 (see Fig. 7 ▶). This is compensated by tilting the first mirror to an angle of α + δ/2 relative to the beam and the second mirror to α − δ/2, so that the exiting beam is again horizontal. Note that δ/2α ≃ 0.01 and hence this readjustment has no significant impact on the reflectivity of the mirrors.

The two mirrors were fabricated by WinlightX, Pertuis, France (http://www.winlightx.com) from single-crystal silicon instead of the more conventional material, fused silica. The main reason for this choice was the fact that the thermal conductivity of silicon is 100 times greater than that for silica, thereby minimizing the formation of thermal bumps when operating in pink-beam mode (*i.e.* bypassing the DCM) for possible future micro-Laue experiments. The mirrors’ reflecting surfaces consisted of 7 mm-wide stripes of Rh coating, bare Si, and Pt coating (coating thickness of 55 nm and 37.5 nm, respectively), separated from each other by 4.5 mm. The useful mirror length was 400 mm and the mirror cross section 40 mm × 40 mm. The tangential slope error was specified to be better than 0.30 µrad (RMS). Each stripe is accessed by translation of the entire mirror chamber in the *x*-direction by ±11.5 mm (see Fig. 7 ▶).

The critical angle for total external reflection, α_c_, is inversely proportional to the photon energy, and proportional to the square-root of the electron density. As is often observed, the actual densities of the sputtered Rh- and Pt-coating stripes are marginally less than for bulk material. These were accurately determined by recording the reflectivity curves of test pieces prepared in the same sputter-deposition run used to make the mirror coatings themselves, and at three different photon energies. The Rh- and Pt-coatings were found to be 12.0 g cm^−3^ (96.8% dense, 3.142 e Å^−3^) and 20.3 g cm^−3^ (94.9% dense, 4.86 e Å^−3^), respectively. The atomic roughness could also be fit, and yielded 0.25 nm for the Rh surface and 0.50 nm for Pt.

The mirrors should be tilted to close to the critical angle, in order to suppress harmonic contamination as much as possible. However, one should avoid getting too close to α_c_, as the drop in reflectivity at the critical angle is not infinitely abrupt. We therefore chose a value of 85% of α_c_. The reflectivity at the lower and upper limits of the energy ranges used for each stripe is given in Table 5 ▶.

We express this set angle in convenient units as 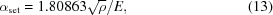
where α_set_ is in degrees, ρ is the electron density in  e Å^−3^, and the photon energy *E* is in keV. This yields 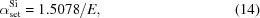


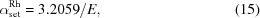


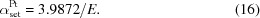
Referring to Fig. 7 ▶, we see there are some constants to the optics geometry. These are:

(i) The vertical offset of the monochromatic beam emerging from the optics compared with that of the incoming pink beam, Δ*y* = *y*1 + *y*2 − *y*3 = +20 mm.

(ii) The horizontal distance between the centres of M1 and M2, *z*3 = 700 mm.

(iii) The horizontal distance between the centres of X1 and M1, *z*0 = *z*1 + *z*2 = 1372 mm.

We now determine expressions for the positions and angles of the four optical components. First, the Bragg angle of X2 is simply 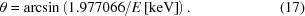
From Fig. 7 ▶, 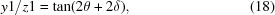


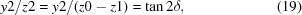


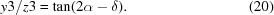
Remembering that Δ*y* = *y*1 + *y*2 − *y*3 leads to 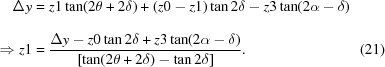
From this, we directly obtain the horizontal distance between the centres of X2 and M1, *z*2 = *z*0 − *z*1, from which *y*1, *y*2 and *y*3 directly follow.

**Figure 11:**
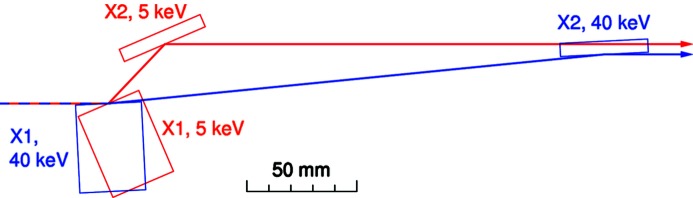
True-scale positions of X1 and X2 at 5 and 40 keV, using the Si and Pt stripes on the mirrors, respectively.

The crystal and mirror positions as a function of photon energy for the three mirror coatings are provided in Fig. 10 ▶, with the coating densities given above. The limits of the positions at the lowest (5 keV, using Si reflection on the mirrors) and highest energies (40 keV, using Pt) are shown in Fig. 11 ▶.

## 7. Performance   

### 7.1. DCM energy resolution   

**Figure 12:**
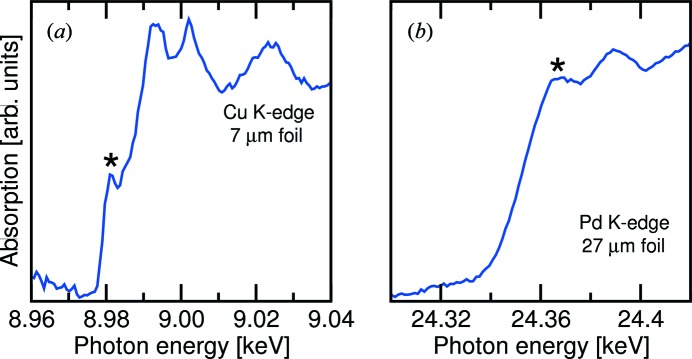
(*a*) Raw *K*-edge XANES spectrum for metallic copper, recorded at the MS beamline using the unfocused undulator beam. From the width of the pre-edge at 8.9813 keV, it was determined that the DCM energy resolution equalled the theoretical value of 1.4 × 10^−4^. (*b*) Raw *K*-edge XANES spectrum for metallic palladium, recorded to test the reliability of the Bragg angles of the two DCM crystals.

Initial calibration of the DCM Bragg angles, plus the DCM resolving power, were tested using an unfocused beam by recording the Cu *K*-edge, which has a well studied pre-edge feature at 8.9813 keV. The raw spectrum, only corrected for the small calibration offset in the Bragg angle, is shown in Fig. 12(*a*) ▶, whereby the pre-edge feature is highlighted with an asterisk. The separation in the valley and peak energies of the pre-edge is 1.7 eV, or 1.9 × 10^−4^ of the edge energy. This high visibility demonstrates that the DCM energy resolution Δ*E*/*E* is at the theoretical value of approximately 1.4 × 10^−4^.

The calibration offsets for the Bragg angles of X1 and X2 determined from the Cu-edge XANES spectrum are only constant if there is insignificant parasitic pitch associated with the relatively large horizontal movement of X2 between low and high photon energies (see Figs. 10 ▶ and 11 ▶). A second Pd-edge XANES spectrum was recorded at 24.35 keV (Fig. 12*b* ▶), from which it was observed that θ was accurate to within 5 × 10^−5^ degrees, and that therefore the absolute accuracy of the set energy lay within the Darwin width of the crystals.

Once the DCM has thermally stabilized after initial cooling of the first crystal to 77 K, the photon energy remains constant to better than 0.3 eV (one part in 10^5^), as was demonstrated by recording at 25 keV a silicon powder pattern every 45 s over 10 h.

### 7.2. Focusing elements   

#### 7.2.1. Primary optics   

As mentioned earlier, vertical focusing is achieved using a flexural hinge-based mirror bender, similar to systems described previously (Rossetti *et al.*, 2002 ▶). The X-ray beam is focused in the horizontal plane by sagittally bending the second DCM crystal X2 (Schulze *et al.*, 1998 ▶).

From the lensmaker equation 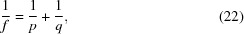
whereby *p* is the source–lens distance and *q* is the lens–image distance, we can establish the required focal lengths for focusing at the PD station, at the SD station and for parallel beam (Willmott, 2011 ▶). For horizontal focusing, *p*_h_ ≃ 20.268 m (the midpoint of the horizontal translation of X2; see Table 4 ▶), while, in the vertical plane, *p*_v_ = 22.214 m, the centre of mirror 2. For focusing at the powder diffractometer, *q*_h,PD_ = 12.529 m and *q*_v,PD_ = 10.583 m, while, at the surface diffractometer, *q*_h,SD_ = 19.819 m and *q*_v,SD_ = 17.873 m. Table 6 ▶ lists in the second and third columns the focal lengths in the vertical and horizontal planes for focusing at the powder diffractometer, the surface diffractometer and for parallel beam.

**Figure 13:**
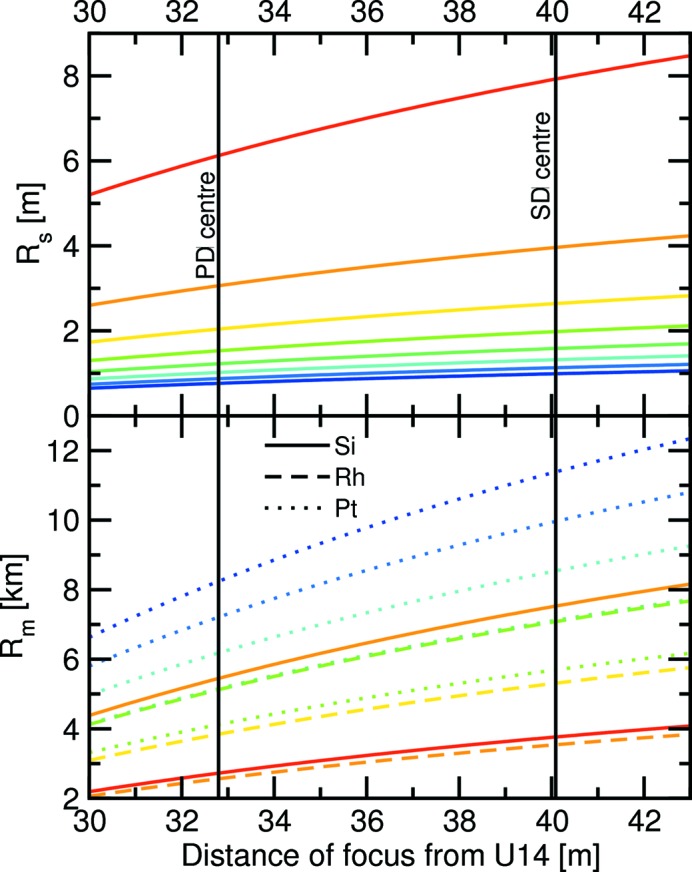
Change in the sagittal and meridional bending radii as the focus is adjusted between 30 and 43 m. The colours of the curves change gradually in 5 keV steps between 5 keV (red) and 40 keV (blue). Because the meridional focus depends on the mirror incident angle, which in turn depends on which stripe is being used (Si, Rh or Pt), these are distinguished by the curves being solid, dashed and dotted, respectively. The positions of the powder diffractometer and surface diffractometer centres are also marked.

The bending radii *R*_s_ and *R*_m_ for sagittal focusing using the second DCM crystal and meridional focusing using the second mirror, respectively, are given by 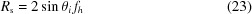
and 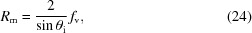
whereby θ_i_ is the incident angle of the X-ray beam on the optical element and is equal to the Bragg angle θ in the case of sagittal focusing using X2; and equal to the mirror incident angle α for vertical focusing using M2. Both θ and α are energy dependent. The limiting values for 5 and 40 keV are listed in Table 6 ▶. The change in the bending radii as the beam focus is adjusted between 30 and 43 m from the centre of U14 is shown in Fig. 13 ▶.

**Figure 14:**
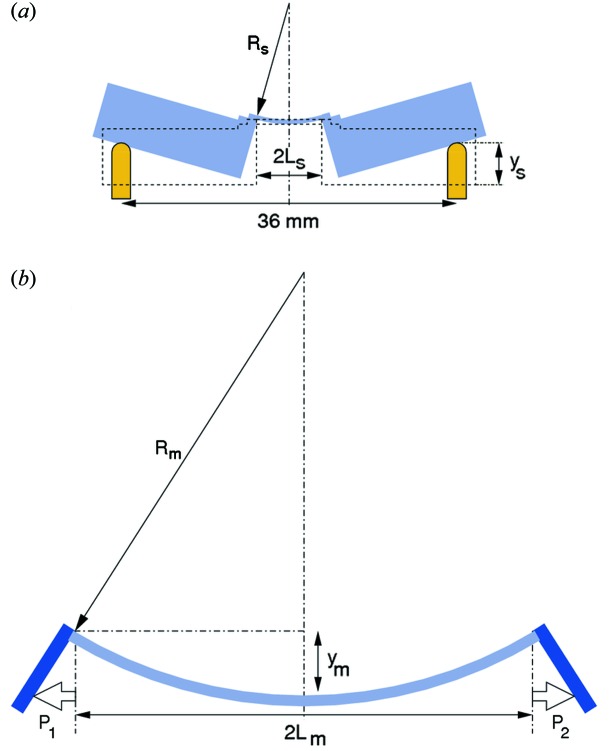
Schematics of (*a*) the sagittal bender geometry and actuators; and (*b*) the meridional bender geometry. The mirror is bent by applying outwards forces on Si blades (shown here in dark blue) attached to the mirror ends, which are pushed outwards by equal amounts *P*_1_ and *P*_2_.

We now consider the necessary range of motion and resolution of the actuators used to bend X2 and M2. We begin by assuming cylindrical focusing. The pertinent geometries are sketched in Fig. 14 ▶. We begin with the simpler configuration of the mirror bender (Fig. 14*b* ▶). From Pythagoras’ theorem,
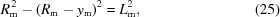
from which one obtains 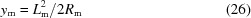
under the valid assumption that *y*_m_/*R*_m_ 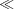 1. Both mirrors in the new optics set-up have a usable length 2*L*_m_ = 400 mm. The maximum value for *y*_m_ is thus at the minimum bending radius, which is required for focusing 5 keV photons at the PD station (*R*_m_ = 2.723 km), and therefore 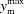 = 7.34 µm.

Differentiation of (26) with respect to *R*_m_ leads to 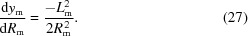
This tells us the change in *y*_m_ (d*y*_m_) for a small change in the meridional bending radius d*R*_m_. What interests us more, however, is the amount we need to move the actuator for a given small change in *q*, the position of the focal spot (*i.e.* d*q*). We differentiate by parts equation (24) with respect to *q* to obtain 
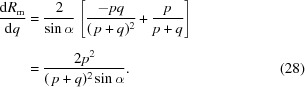
Multiplying (27) with (28), we obtain 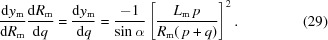
Substituting for *R*_m_ using (24) leads to 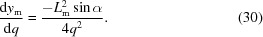
We want to control the focal position with an accuracy of 10 cm. From (30), the smallest change in *y*_m_, d*y*_m_, is required for large *q* (*i.e.* at the surface diffractometer) and for high photon energies (small incident angle α). Under these conditions, control of *q* with an accuracy d*q* = 10 cm therefore requires d*y*_m_ = 5.4 nm, approximately 1400 times smaller than 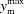.

**Figure 15:**
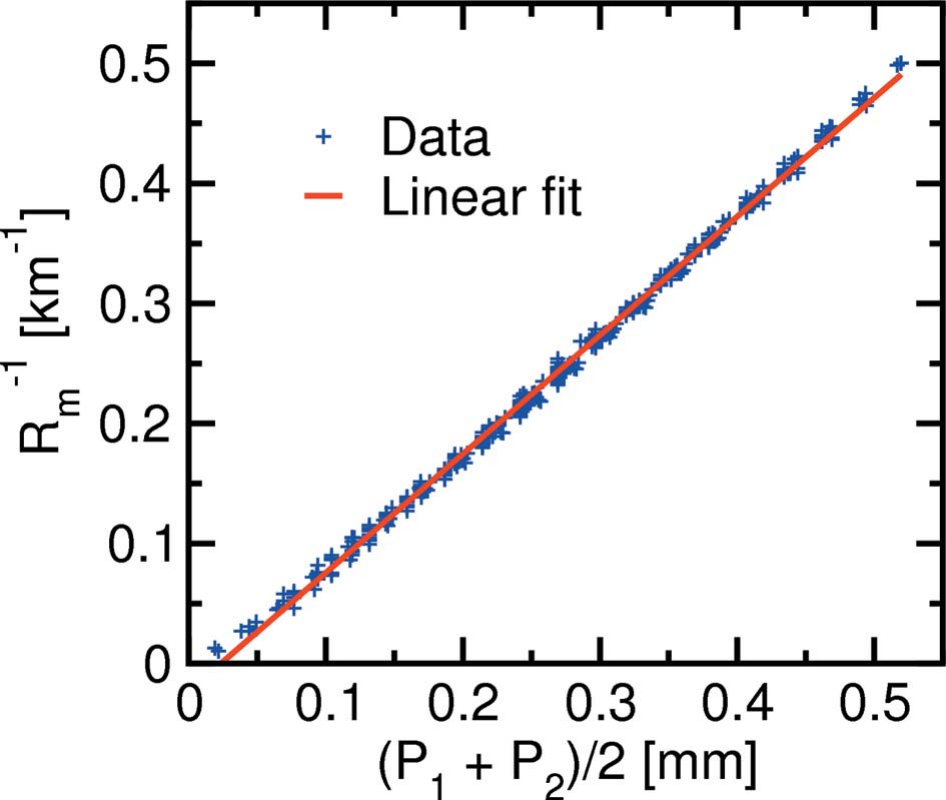
The bending radius of the second mirror measured using a long-trace profiler *versus* the average of the Si-blade actuator positions (*P*_1_ + *P*_2_)/2. The best linear fit is also shown.

Bending mirror 2 was achieved using the flexural hinge system described by Rossetti *et al.* (2002 ▶). Silicon blades, 10 mm-thick, are attached to a rigid clamp system holding the ends of the Si mirror. The bending depth *y*_m_ is proportional to the amount the Si blades are pushed outwards (*P*_1_ and *P*_2_ in Fig. 14 ▶). It is hence clear from equation (26) that the meridional radius of the second mirror, *R*_m_, is inversely proportional to the positions of the Si-blade actuators. This is demonstrated in Fig. 15 ▶ using a long-trace profiler. Driving both motors to push out the blades by *P*_1_ = *P*_2_ = 0.5 mm was shown to induce the minimum bending radius of approximately 2 km.

X2 is sagittally bent by applying equal forces on four actuators (two pairs, each pair being separated by 36 mm). From Fig. 14(*a*) ▶, we see that the height of the four actuators, *y*_s_, above that in the flat configuration, is composed of two parts: *y*_s1_, due to the bending of the thinned central part of width 2*L*_s_ = 7 mm, and *y*_s2_, produced by the tilting of the rigid blocks. *y*_s1_ is derived in the same manner as *y*_m_ in equation (26), so that 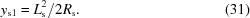

*y*_s2_ can be simply derived by using the principle of similar triangles, 
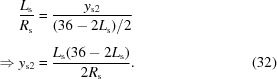
Combining (31) and (32) we obtain 
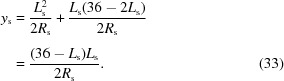
The maximum travel of the sagittal actuators is therefore approximately 75 µm.

Using the same approach as above for meridional focusing, and referring to (33) and (23), leads to 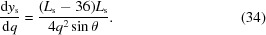
Because of the larger divergence in the horizontal plane compared with that in the vertical plane, it is unnecessary to control the focal point to better than approximately 1 m. Control of *q* to this amount thus requires (at low photon energies at the surface station) a minimum step size in *y*_s_ of 180 nm. In the present set-up, the pitch of the sagittal actuators is 250 µm and the motors have a resolution of 1000 steps per turn, sufficient for all conditions except the lowest-energy experiments at the SD station.

**Figure 16:**
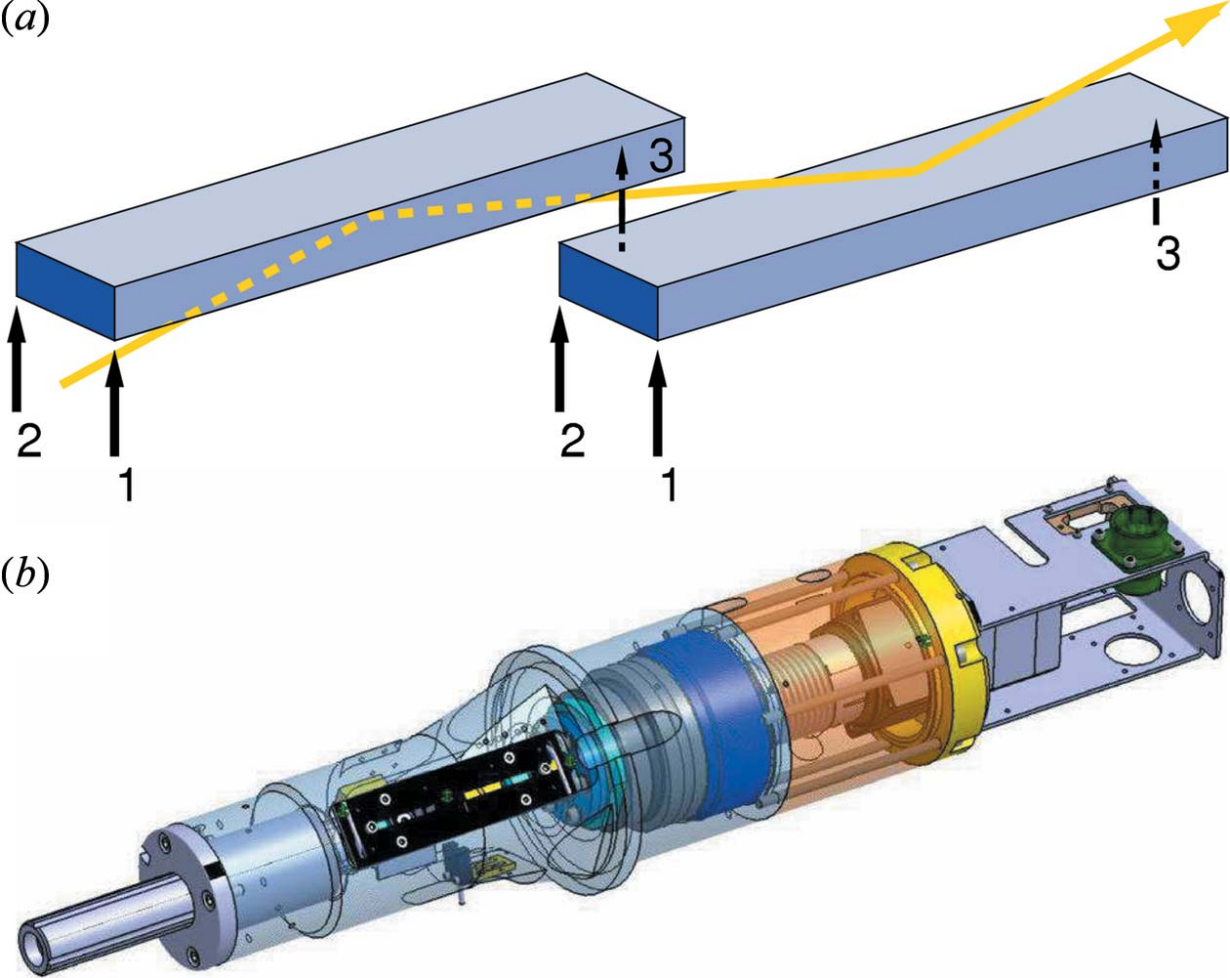
(*a*) Schematic of the linear drive set-up for tilting and rolling the X-ray mirrors. (*b*) Detail of the drive design.

Both mirrors can be dynamically tilted (‘pitch’ motion), a requirement set by the change in incident angle with photon energy [Fig. 16(*a*) ▶ and equations (14)–(16)]. We specified the tilt accuracy to be equal to or better than 10 µm in the vertical position at the SD station. This imposes an accuracy of better than 80 nm for the linear positions of the 3 × 2 linear drives used to tilt the mirrors. These drives, adapted in-house from an earlier design from DESY, Hamburg, were found to have an accuracy of better than 50 nm when operated in open loop. Under normal operating conditions, the absolute positions were controlled using encoders with 50 nm resolution. A sketch of the linear drive design is shown in Fig. 16(*b*) ▶.

The best focal spots obtained to date using the primary optics have been 80 µm × 30 µm at the PD station and 130 µm × 40 µm at the SD station.

#### 7.2.2. FZP microfocusing   

**Figure 17:**
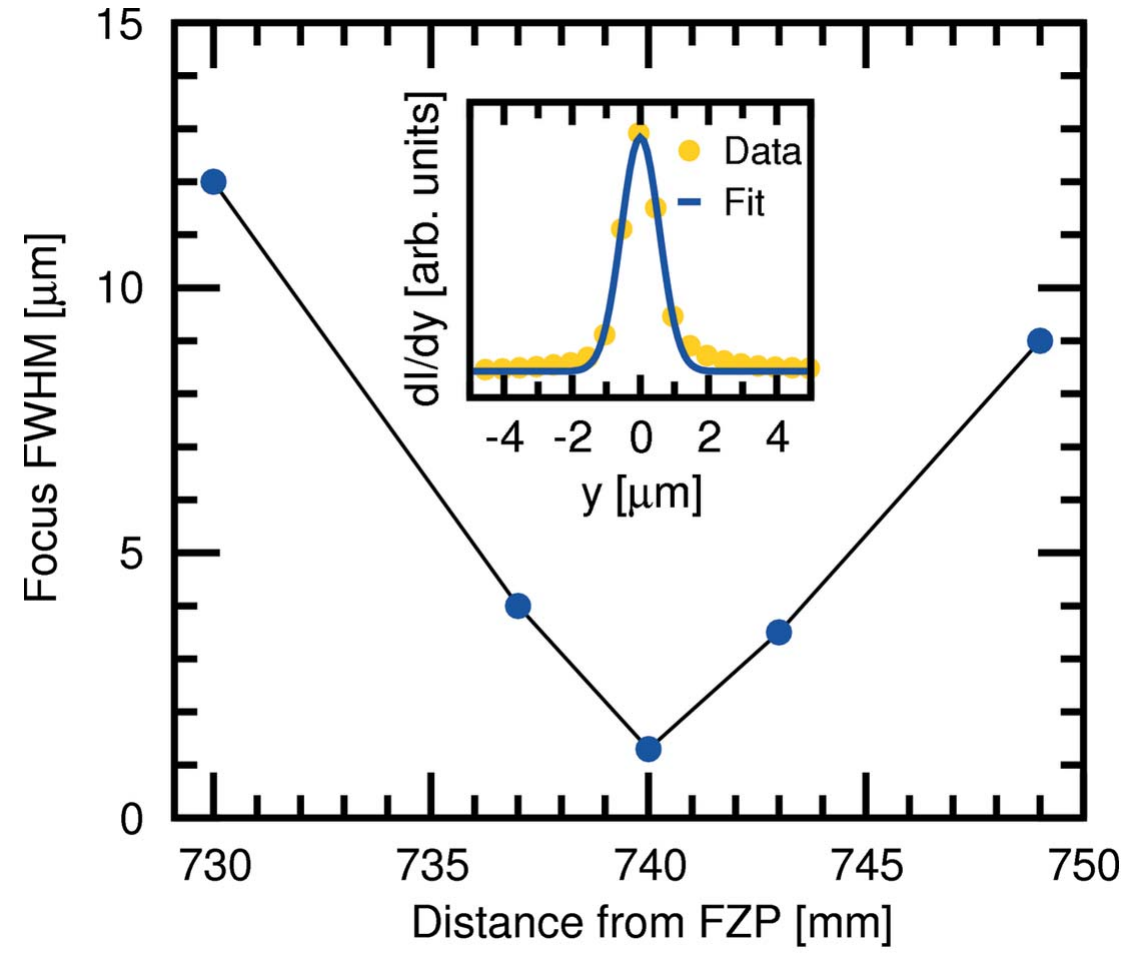
Change in the focused FWHM beam diameter as a function of distance downstream from the FZP. The tightest focus at 740 mm has a FWHM width of 1.32 µm (see inset).

Fresnel zone plates (FZPs) fabricated in-house by the Laboratory of Micro- and Nanotechnology can be employed to demagnify the source by approximately a factor of 50. The first FZP, made of 0.82 µm-thick gold Fresnel zones on a Si_3_N_4_ membrane (outer zone width of 100 nm and diameter of 1 mm) was tested at the SD station using 9 keV radiation. The FZP nominal focal length is 725.9 mm. The source–sample distance at the SD station is 40.087 m (see Table 4 ▶); hence, using the lensmaker’s equation, we would expect the tightest focus at a distance of 739.5 mm downstream from the FZP. The experimentally determined smallest focus was found to be at this position to within 0.5 mm, had a FWHM of 1.32 µm (Fig. 17 ▶) and transmitted a flux of 5 × 10^10^ photons s^−1^, equating to a flux density of 3.7 × 10^16^ photons s^−1^ mm^−2^, approximately 12 times higher than the best focus at SD using only the primary optics. The FWHM beam divergence using this FZP is 1.35 mrad (0.077°).

## 8. Endstations   

### 8.1. Powder diffraction   

#### 8.1.1. Mythen II microstrip detector   

**Figure 18:**
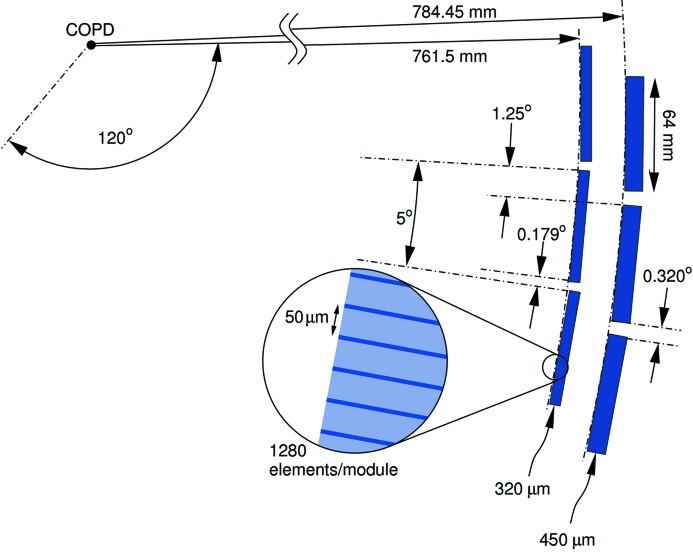
Schematic of the most important geometrical parameters of the upgraded Mythen II detector at the PD station. COPD = centre of the powder diffractometer.

The Mythen II microstrip detector has become the workhorse of the PD station (Bergamaschi *et al.*, 2010 ▶) since its installation in 2007. The Mythen detector has recently undergone a significant upgrade, in which a second layer of detector modules was installed. Each layer covers an angle of 120° and consists of 24 modules. The geometry of this new set-up is shown in Fig. 18 ▶. The radii of the circles tangential to the layer surfaces are 761.5 mm (inner layer, Si-module thickness 320 µm) and 784.45 mm (outer layer, thickness 450 µm). The modules of both layers are 1280 channels wide (channel separation of 50 µm) and are repeated every 5°. The outer layer is offset relative to the inner layer by approximately 1.25°, in order to provide a gapless angular response, thereby obviating the need to move the detector to cover the small gaps between modules (0.179° and 0.320° for the inner and outer layer, respectively). The minimum readout time (*i.e.* dead-time when operating in the non-symmetric mode; see below) is 90 µs for 4-bit low-dynamic-range data.

There are further advantages in having the second layer. Firstly, the thicker outer modules are more sensitive to high-energy X-rays. Secondly, rate corrections for intense (*e.g.* Bragg-peak) signal can be avoided or at least reduced, since the data on the inner layer can be discarded, because the statistics for the signal from the outer layer are already high.

The intrinsic resolution of Mythen II in 2θ is arctan(0.05/761.5) = 0.00376°. The effective resolution, however, is determined by the sample or illumination size, such that, for example, the resolution of data acquired from a homogeneously illuminated capillary of 200 µm diameter is 0.0188° (Gozzo *et al.*, 2004 ▶, 2010 ▶). In the large majority of cases, this is more than sufficient for PD applications, be they structural solutions, profile/full-pattern analysis or total scattering. Only in rare cases in which the sample intrinsic line broadening is close to line-profile standards is the resolution using Mythen II inadequate. In particular, Mythen II has been shown to be perfectly sufficient for full structural determination (Bruni *et al.*, 2011 ▶). Moreover, the parallel data acquisition over large angular ranges means that (*a*) time-resolved studies on a millisecond timescale or shorter and (*b*) the complete exclusion of radiation damage, are possible with Mythen II, which would be impossible using analyzer detectors (Bergamaschi *et al.*, 2010 ▶; Fadenberger *et al.*, 2010 ▶; Willmott, 2011 ▶).

The upgrade of Mythen II has also included operational improvements. It can now be used in a symmetrical configuration (±60°), whereby data acquisition from one half is delayed with respect to the other, for dead-time-free measurements. When operating continuously and with the maximum dynamic range of 24 bits (16 Mcounts), the maximum data acquisition rate for the full 120° is 20 frames s^−1^. This scales inversely with the number of modules used and the dynamic range. Lastly, the on-board memory allows for storage of data up to several thousand frames per burst before data must be transferred to file.

#### 8.1.2. SAXS/WAXS facility   

**Figure 19:**
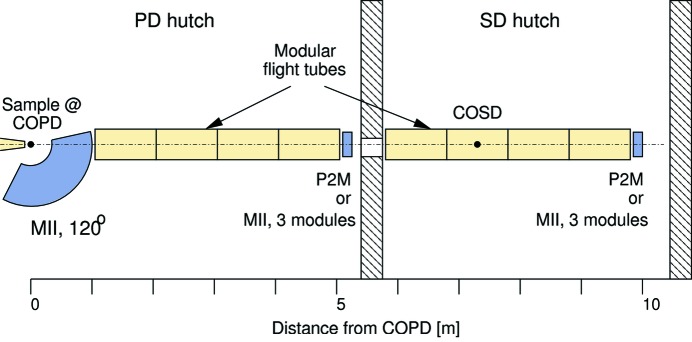
Schematic of the possible set-ups for SAXS/WAXS experiments at the MS beamline. A transparent window in the housing of the 120° Mythen II detector (MII, 120°, used for WAXS data) reduces the dead region to zero (or the beam stop size) between scattering up and down. The SAXS data can either be recorded in the PD hutch using a Pilatus 2M detector [P2M, area 253 mm (h) × 288 mm (v)] or three Mythen II modules (length ≃ 192 mm). Extension to sample–detector distances as large as 10 m is possible if the P2M is placed in the SD hutch. The evacuated or He-filled flight tubes are constructed from modular 1 m-long units. COSD = centre of the surface diffractometer.

A new combined small-angle/wide-angle X-ray scattering (SAXS/WAXS) set-up has been designed at the MS beamline, with a view to investigating phase changes and catalytic processes on micrometre- and submicrometre-sized powder systems. Fig. 19 ▶ shows the possible configurations. WAXS data are recorded using the large Mythen II microstrip detector. A crystalline transparent window at one edge of the WAXS detector housing allows the SAXS signal to propagate with zero dead region on-axis (in reality, the dead region is determined by a beam stop directly in front of the SAXS detector). The sample–SAXS detector distance can be varied, according to the length of the flight tube in between, which consists of modular 1 m sections. The choice of the SAXS detector depends on the needs of the experiment; the three-module Mythen II detector provides better resolution in reciprocal space, but only in one dimension, and has a smaller maximum scattering vector *Q*_max_. Conversely, the Pilatus 2M detector is 50% larger and can therefore in principle detect features that are correspondingly smaller, although the largest objects that can be resolved are three times smaller than those studied with the Mythen modules.

The largest scattering vector *Q*_max_ and best resolution Δ*Q* are listed for both WAXS and SAXS at a photon energy of 10 keV in Table 7 ▶.

#### 8.1.3. Other equipment   

**Figure 20:**
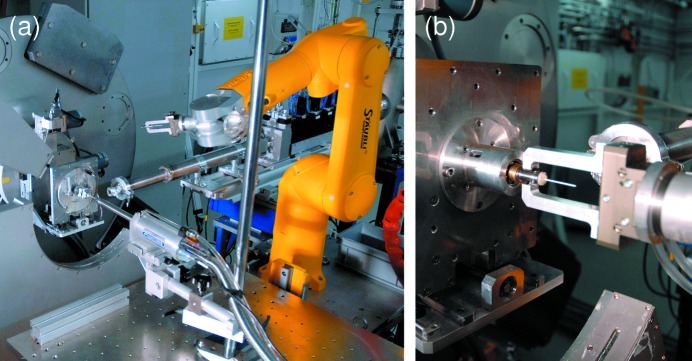
The robotic Stäubli TX60L sample changer at the PD station. (*a*) The robot arm in relation to the powder diffractometer. (*b*) Close-up of the sample grabber and magnetic cone mounting system.

Several sample environments are available for users at the PD station, listed in Table 8 ▶. In addition, a robotic sample changer (Stäubli, model TX60L) allows rapid and remote sample exchange, particularly useful for experiments in which many samples need to be investigated (Fig. 20*a* ▶). Most of such experiments are performed using the Mythen detector (and not the crystal analyzer), hence it is imperative that the sample capillary is mounted as coaxially as possible with the diffractometer axis, as the sample holders used by the robot system allow no adjustment of the sample orientation (Fig. 20*b* ▶). A system is presently under development to simply and rapidly mount capillaries of all diameters concentrically on the magnetic cone sample holders.

### 8.2. Surface diffraction   

#### 8.2.1. Detectors   

The two-dimensional photon-counting Pilatus 100k detector has been permanently installed at the SD station since 2005 (Schlepütz *et al.*, 2005 ▶, 2011 ▶). The original graphite-crystal analyzer has been removed. In the present set-up, the sample–detector distance of 1140.8 mm and pixel size of 172 µm × 172 µm provides an angular resolution of 0.00862° (Δ*Q* = 7.6 × 10^−4^ Å at 10 keV).

At the time of writing, steps are being taken to replace the Pilatus 100k detector with the next-generation ‘Eiger 500k’ detector (Dinapoli *et al.*, 2010 ▶), consisting of 1024 (h) × 512 (v) pixels, each pixel being 75 µm × 75 µm in size. At the same sample–detector distance as presently used, this will provide a resolution of Δ*Q* = 3.3 × 10^−4^ Å at 10 keV. As discussed in §9.2.1, extending the sample–detector distance to 2 m will enhance the resolution to approximately 1.9 × 10^−4^ Å.

The ‘standard’ Eiger module is water-cooled. Although this poses no problem for experiments in which the detector is stationary, such as in SAXS, water-cooling is difficult to implement at the surface diffractometer, as the detector module is mounted on the rotatable detector arm. The Eiger module being developed for the SD station is therefore air-cooled.

#### 8.2.2. Environmental chambers   

For robust sample surfaces, in particular those of many perovskites and related complex metal-oxides, it is only necessary to maintain an oxygen/water partial pressure below approximately 1 mbar in order to avoid radiation damage. For such samples, two very simple and easy-to-use systems are available. The first is a 50 mm-diameter 0.5 mm-thick beryllium dome, pumped *via* a Teflon tube with a turbomolecular pump to approximately 10^−2^ mbar. The alternative to this is an 80 mm-diameter 25 µm-thick thermoformable polyimide dome filled with helium gas. This system has the important advantage over the Be dome that there is essentially no diffuse scattering of the incident and exit beam by the dome walls, and, because the plastic is amorphous, polycrystalline Debye–Scherrer cone signal from the dome is entirely absent.

**Figure 21:**
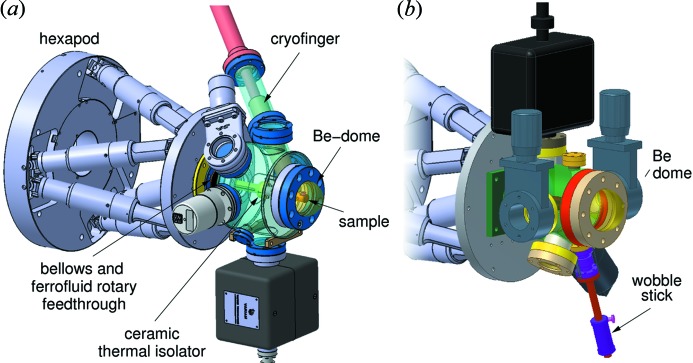
(*a*) UHV cryostat chamber for experiments in which the sample needs to be cooled to as low as 4.2 K. (*b*) UHV ‘baby chamber’ for experiments requiring vacuum below 10^−9^ mbar.

Recently, a new UHV cryostat has been designed and constructed for SD experiments. The lowest achievable temperature is 13 K. This replaces the previous system, which was designed for bulk single-crystal diffraction experiments and was thus far from optimal for SD experiments. The sample movements in the new design (*i.e.* rotation of the sample about its surface normal, and fine adjustment of the surface orientation using the hexapod) are decoupled from the vacuum vessel (which is fixed to the optical table on the surface diffractometer) using bellows and a ferrofluid rotary feedthrough (see Fig. 21*a* ▶). A rod is attached to the vacuum side of this feedthrough, which is in turn connected *via* a ceramic spacer to the cooled sample holder, made from copper. The spacer thereby thermally isolates the sample mount from the rest of the chamber, allowing for rapid thermal equilibration and mechanically stable conditions. The sample holder is connected *via* copper braids to the Janis ST-400-1 UHV continuous-flow cryostat. As well as cooling, the cryostat can be heated to 500 K. All vacuum seals are UHV-compatible conflat flanges. The ultimate pressure lies in the mid 10^−10^ mbar range.

For experiments requiring only the best vacuum conditions, a second ‘mini chamber’ is available, based on the design of Lee & Zegenhagen (2006 ▶). Samples can be inserted and removed by attaching a vacuum load lock onto a side port and are held using screws that are manipulated using a wobble stick (see Fig. 21*b* ▶). The ultimate pressure lies in the low 10^−10^ mbar range.

Lastly, an *in situ* thin-film growth chamber using pulsed laser deposition is also available, as described in detail elsewhere (Willmott *et al.*, 2005 ▶).

## 9. Initial results   

### 9.1. Powder diffraction   

**Figure 22:**
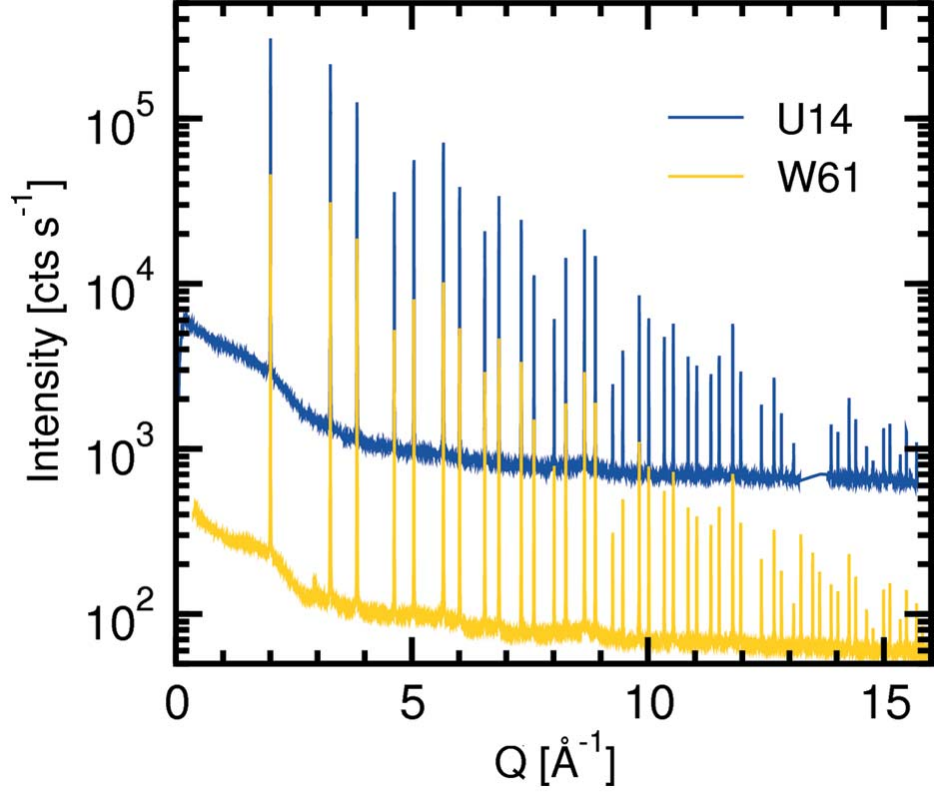
Powder patterns of Si powder recorded using wiggler radiation (gold) and undulator radiation (blue). The approximately factor-of-two larger noise in the latter is due to the fact that the exposure time was four times shorter than in the former.

Standard Si-PD patterns were recorded at 20 keV using the Mythen II detector, under comparable conditions [with regards to the capillary diameter (300 µm), filling fraction, length of capillary exposed, and vertical beam divergence], and using parallelized radiation from the W61 wiggler in 2010 and naturally divergent radiation from the U14 undulator in 2012. In the former the capillary was exposed for 160 s, while in the latter the exposure time was 40 s. The results are compared in Fig. 22 ▶. The count rate is approximately eight times larger when using undulator radiation. This corresponds very well to the theoretically expected ratio of the beam cross-sections at the powder station, assuming similar total flux from U14 and W61 at this energy.

### 9.2. Surface diffraction   

**Figure 23:**
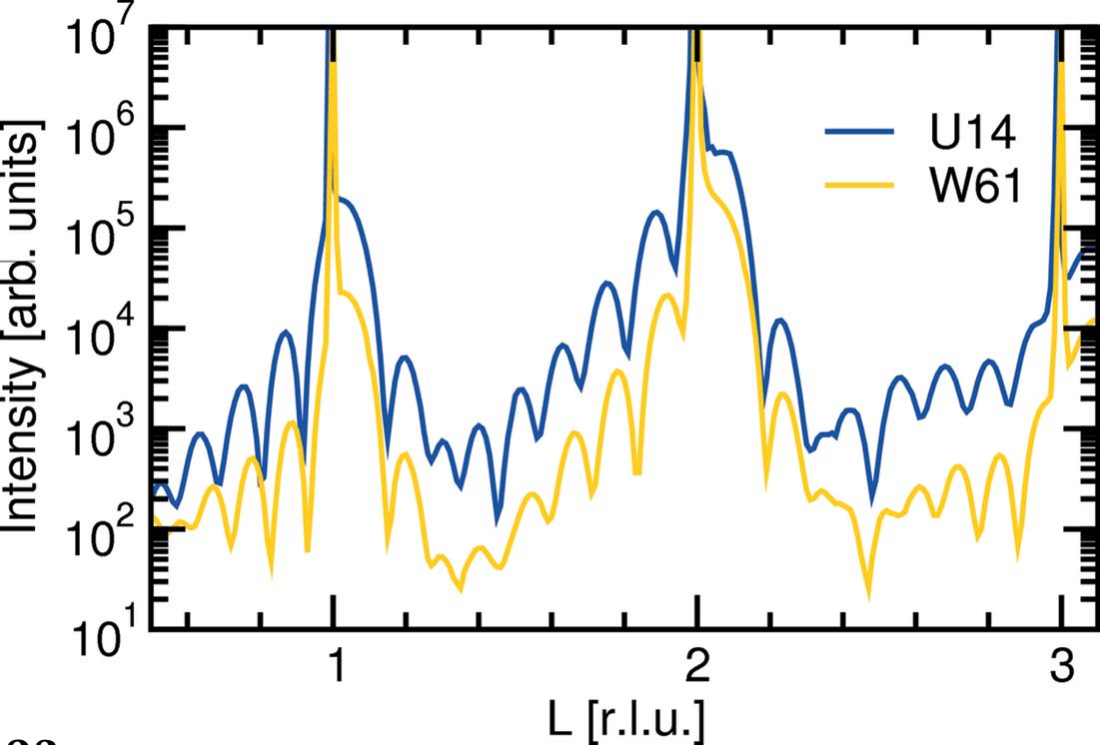
The (00*l*) crystal-truncation rod of the same LaNiO_3_ thin film grown on SrTiO_3_ using the W61 wiggler and the U14 undulator.

A comparison of the (00*l*) crystal-truncation rod (CTR) of the same LaNiO_3_ thin-film sample using wiggler and undulator radiation at 15.5 keV is shown in Fig. 23 ▶. A similar increase in signal intensity (after background subtraction) of approximately a factor of ten was obtained. Marginal differences in the details of the CTR profiles can be explained by the footprint of the undulator radiation being approximately a factor of five smaller than that of the wiggler source, plus the fact that the LaNiO_3_ sample was two years older when the U14-CTR was recorded.

#### 9.2.1. Coherent X-ray diffraction imaging   

Coherent X-ray diffraction imaging (CXDI) in the Bragg geometry is an emerging quantitative technique for imaging the internal defect structure of nano- and micro-crystalline objects and materials (Robinson & Harder, 2009 ▶) and extended samples (Robinson *et al.*, 2005 ▶; Le Bolloc’h *et al.*, 2005 ▶). Phase retrieval and inversion of the oversampled intensity distribution around a Bragg peak (Sayre, 1952 ▶) allows one to obtain the electron density and relative displacements (*i.e.* strain fields) with sub-ångström resolution of the atoms from their ideal bulk-like positions (Pfeifer *et al.*, 2006 ▶).

At present the coherence volume available at the MS beamline is of the order of ξ_T,h_ = 10 µm, ξ_T,v_ = 100 µm, ξ_L_ = 1 µm. The 2 + 3 circle surface diffractometer allows sufficient degrees of freedom to access and map in three dimensions individual Bragg reflections. The secondary optic FZP described in §7.2.2 allows micrometre focusing.

At the end of November 2012, the first CXDI experiments were performed at the MS beamline. Two modes of operation were tested: one in which the transmitted coherent flux was selected from the unfocused beam using a coherence defining slit [20 µm (h) × 100 µm (v)], the second with the FZP optics described above.

**Figure 24:**
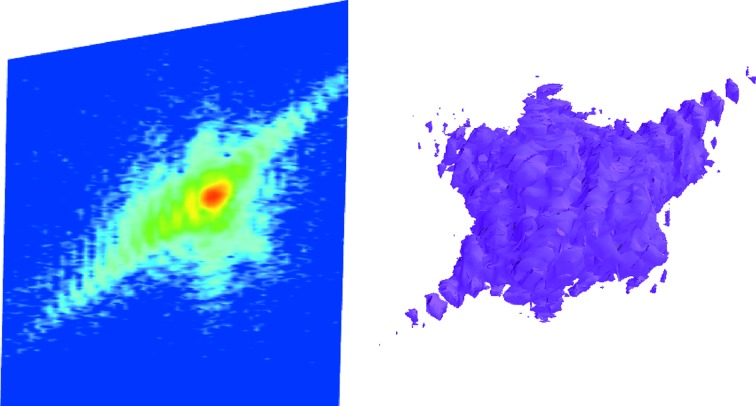
Left: a plane in reciprocal space containing the (111) Bragg peak of a 500 nm gold crystal. Right: the corresponding isosurface of the local three-dimensional scattering intensity distribution.

In Fig. 24 ▶, the source coherence was confirmed in the diffraction pattern of a 500 nm gold crystal by the high visibility of the fringes. The example shown was collected at 9 keV using the FZP optics; 100 min total exposure yielded 1.45 × 10^7^ photons in the diffraction pattern, with a peak intensity of 6.8 × 10^4^ photons.

At present the detector–sample distance and pixel size determine the upper limit on the crystallite size to approximately 500 nm, while the lower limit is set by the flux density; in the unfocused mode, 300 nm is approximately the lower limit, given the increased exposure time required to count statistically significant scatter. Below this size, focusing optics are mandatory. Implementation of the Eiger detector and an increased detector-arm length will relax the upper limit considerably to closer to two micrometres, at which point the longitudinal coherence length will become the limiting factor.

In the long term, multiple Bragg reflections from the same crystal would provide the full three-dimensional strain tensor (Newton *et al.*, 2010 ▶) and could be exploited to characterize the internal structure of devices *in situ*, during synthesis, and operation in a working environment.

## 10. Concluding remarks   

The Materials Science beamline has been upgraded with an undulator X-ray source having the smallest ever reported magnet-pole periodicity of 14 mm to be routinely used in synchrotron storage rings. This allows photon energies up to nearly 40 keV to be accessible from the medium-energy storage ring of the SLS. New X-ray optics suited to the power density and beam cross-section of the source were designed and installed. The beamline performs close to theoretical values up to approximately 25 keV, dropping to approximately 50% thereafter.

The improved brilliance has resulted in an order-of-magnitude improvement in diffraction intensity at both the PD and SD stations. The increased transverse coherence lengths of the undulator radiation has opened up the possibility of performing coherent diffraction imaging in the Bragg geometry.

## Figures and Tables

**Table 1:** SLS specifications

Parameter	Value
Storage-ring energy	2.4GeV
Lorentz function	4697
Circumference	288m
Electron current	400mA (top-up operation)
Horizontal emittance	5.5nmrad
Horizontal beta function	1.38mrad^1^
Vertical emittance	5pmrad
Vertical beta function	1.0mrad^1^
Electron-beam size (FWHM)	204m (h) 5.3m (v)

**Table 2:** Materials Science beamline specifications

Parameter	Value
Photon energy range	540keV
Flux at 12keV	>10^13^photons s^1^
Energy resolution *E*/*E*	1.4 10^4^
Focus at SD station (1:1)	Better than 200m (h) 30m (v)

**Table 3:** U14 CPMU specifications compared with those of the W61 wiggler previously installed at the MS beamline and the U19 undulator installed at the PX1 beamline of the SLS

Parameter	U14	W61	U19 at PX1
Overall length (m)	1.680	1.906	1.82
Period length (mm)	14	60.5	19
Number of poles (2*N*)	240	63	192
Power output (W)	3000	5700	2600
Beam divergence [FWHM h (rad) v (rad)] at 12keV	144 16.5	2500 230	135 25
Minimum magnetic gap (mm)	3.8	8	4.5
Maximum deviation parameter *K*	1.46	8.6	2.46
Beam size at DCM [FWHM h (mm) v (mm)]	2.9 0.33	49 4.5	2.65 0.49
Power on first DCM crystal (W)	157	733	137
Maximum field (T)	1.26	1.84	1.39
Critical energy (keV)	4.8	7.0	5.3
Flux at 12keV (photons s^1^)	2 10^13^	10^13^	2 10^12^
Best focus (1:1) [FWHM (m)]	80 30	450 300	

**Table 4:** Positions of important beamline components with respect to the centre of the U14 undulator

Component	Distance (m)	Comment
U14 CPMU	0	Power output = 3000W
Front-end mask	8.245	Protection for XBPMs
XBPM1	8.580	Blade (white) beam-position monitor
XBPM2	11.624	Blade (white) beam-position monitor
Diaphragm 1	12.510	Water cooled; 4.9mm 2.0mm; 390rad 160rad; transmitted power = 1090W
Diaphragm 2	17.054	Water cooled; 4.6mm 1.2mm; 270rad 70rad; transmitted power = 334W
CVD diamond filter	17.636	120m thick, water cooled; absorbs soft X-rays
CVD diamond window	17.851	80m thick, water cooled; vacuum protection for front-end; transmitted power = 157W
Diamond filter set	18.855	0, 50, 100, 200, 400m thick; water cooled
Si filter set	18.980	0, 100, 200, 400, 800m thick; water cooled
Beam-defining slits	19.16919.319	Tungsten, water cooled; accuracy = 1m
Quadrant BPM 1	19.390	9.6mm 3.6mm, suitable for pink beam
DCM crystal 1	20.142	Centre of Si(111), liquid-N_2_ cooled
DCM crystal 2	20.16820.367	Centre of Si(111); sagittal focusing system (Schulze *et al.*, 1998 ▶)
Quadrant BPM 2	20.690	9.6mm 3.6mm, not suitable for pink beam
Mirror 1	21.514	Centre of Si flat mirror, faces down; adjustable mirror tilt
Mirror 2	22.214	Centre of Si-bendable mirror for vertical focusing, faces up; adjustable mirror tilt
Si_3_N_4_ window	23.053	1m thick; two apertures ofmm diameter for pink beam (height = 1400mm) and monochromatic beam (height = 1420mm); separates UHV from low-quality vacuum upstream
Quadrant BPM 3	23.326	9.6mm 3.6mm, not suitable for pink beam
Bremsstrahlung blocker	30.485	Protection against Bremsstrahlung originating in the storage ring; 30mm Cu + 150mm W
Powder diffractometer	32.797	Experimental hutch 1, demagnification 0.6
Surface diffractometer	40.087	Experimental hutch 2, demagnification 0.98

**Table 5:** Reflectivity *R* at 85% of the critical angle _c_ for the three stripes of the two mirrors at their lower and upper limits of the photon energy (given inkeV)

Material	*E*_min_/*R*	*E*_max_/*R*
Si	5/0.835	10/0.950
Rh	10/0.833	20/0.943
Pt	20/0.760	40/0.931

**Table 6:** Focal lengths (in metres) in the horizontal and vertical planes for three modes of operation: focusing at PD, at SD and parallel beam; plus their associated sagittal bending radii (cm), and meridional bending radii (km) at the two limits of the photon-energy range of the MS beamline

Mode	*f*_h_	*f*_v_	*R*_s_ (5keV)	*R*_s_ (40keV)	*R*_m_ (5keV)	*R*_m_ (40keV)
PD	7.743	7.168	612.34	76.54	2.723	8.240
SD	10.020	9.904	792.41	99.05	3.763	11.386
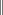 beam	20.268	22.214	1602.8	200.4	8.440	25.537

**Table 7:** Largest scattering vectors *Q*_max_ and best resolution *Q* for different configurations in the SAXS/WAXS set-up at 10keV (see also Fig. 19 ▶)

Detector/distance (m)	*Q*_max_ (^1^)	*Q* (^1^)
MII, 120 (WAXS)/0.761	8.78	1.66 10^4^
P2M (SAXS)/1.2	1.191†	7.21 10^4^
MII, 3 modules (SAXS)/1.2	0.803†	2.10 10^4^
P2M (SAXS)/5	0.292†	1.74 10^4^
MII, 3 modules (SAXS)/5	0.194†	5.07 10^5^
P2M (SAXS)/10	0.044‡	8.72 10^5^
MII, 3 modules (SAXS)/5	0.044‡	2.53 10^5^

† Assumes direct beam at edge of detector.

‡ Limited by the opening angle (2 = 0.50) of aperture between PD and SD hutch 5.6m downstream from the centre of the powder diffractometer (COPD).

**Table 8:** List of available sample environments at the PD station

Equipment	Operating range	Comments
Janis cryostat	4.5300K	Capillary samples only
Oxford cryojet	90500K	
Heat gun	3001300K	
Stoe furnace	3001700K	Capillary samples only
MRI furnace	3002000K	Flat-plate samples only
Membrane diamond-anvil cell	0.120 GPa	0.5mm-diameter culets
